# Inflammation, Stress Response, and Redox Dysregulation Biomarkers: Clinical Outcomes and Pharmacological Implications for Psychosis

**DOI:** 10.3389/fpsyt.2017.00203

**Published:** 2017-10-25

**Authors:** Stefania Schiavone, Luigia Trabace

**Affiliations:** ^1^Department of Clinical and Experimental Medicine, University of Foggia, Foggia, Italy

**Keywords:** biomarkers, psychosis, oxidative stress, first psychotic episode, high risk state

## Abstract

In recent years, several studies claiming the finding of a specific biomarker for the identification of the “high-risk state” to develop psychosis, first psychotic episode, as well as the prediction of the individual response to antipsychotics have been published. Together with genetic reports, numerous publications in this field have been focused on inflammation and stress response blood biomarkers, as well as on indicators of redox dysregulation. In this review, we focus on human studies found in PubMed from January 1^st^ 2010 to January 31^st^ 2017, describing the clinical use of these biomarkers to detect the “premorbid” psychotic state and early phases of the disease. Their pharmacological implications in predicting and monitoring the individual response to antipsychotic medication is also discussed.

## Introduction

Current literature includes several definitions of *“biological markers”* or *“biomarkers.”* In 1998, the National Institutes of Health (NIH) Biomarkers Definitions Working Group was constituted and, in 2001, the following definition of biomarker was published: *“a characteristic that is objectively measured and evaluated as an indicator of normal biological processes, pathogenic processes, or pharmacologic responses to a therapeutic intervention*” (1). In the same year, the International Program on Chemical Safety proposed another definition of biomarker: “*any substance, structure, or process that can be measured in the body or its products and influence or predict the incidence of outcome or disease.”* Both, these two definitions were established starting from a previous statement of the World Health Organization, describing a valid biomarker as: “*almost any measurement reflecting an interaction between a biological system and a potential hazard, which may be chemical, physical, or biological. The measured response may be functional and physiological, biochemical at the cellular level, or a molecular interaction*” (http://www.inchem.org/documents/ehc/ehc/ehc155.htm).

Given the crucial role that biomarkers can play in allowing and improving drug development progression, the economic investments and academic efforts in last decades were highly focused on the identification of valuable biomarkers for the most health impacting medical conditions. Indeed, over $ 2.5 billion were recently granted by the NIH to research proposals aimed to the identification of novel biomarkers. However, this did not result in the expected translation into clinical practice, especially with respect to biological markers identified by proteomic studies (2).

Together with genetic studies, research in the field of psychosis has focused on the identification of inflammation-related biomarkers and mediators of the stress response in peripheral blood samples. Another recent and growing field of research on possible clinic and therapeutic use of specific biomarkers in this mental disorder has been focused on the imbalance between free radical production and the antioxidant system functioning, resulting in redox dysregulation and oxidative stress. In this review, we analyzed studies on humans, found on PubMed from January 1^st^ 2010 to January 31^st^ 2017 and focused on the clinical use of inflammation and stress response blood biomarkers, as well as redox dysregulation biomarkers, in detecting the “premorbid” psychotic state and early phases of the disease. Their pharmacological implications in predicting and monitoring the individual response to antipsychotic medication are discussed.

## Literature Search Strategy

The literature source for the writing of this review was represented by papers present in PubMed from January 1^st^ 2010 to January 31^st^ 2017, found by using the following keyword combinations: biomarkers AND psychosis; biomarkers AND first psychotic episodes; biomarkers AND psychosis high risk state; biomarkers AND antipsychotics; biomarkers AND psychosis AND inflammation; biomarkers AND psychosis AND stress-response; biomarkers AND psychosis AND cortisol; biomarkers AND psychosis AND oxidative stress; biomarkers AND psychosis AND redox dysregulation; biomarkers AND first psychotic episode AND inflammation; biomarkers AND first psychotic episode AND stress-response; biomarkers AND first psychotic episode AND cortisol; biomarkers AND first psychotic episode AND oxidative stress; biomarkers AND first psychotic episode AND redox dysregulation; biomarkers AND psychosis high-risk state AND inflammation; biomarkers AND psychosis high risk state AND stress-response; biomarkers AND psychosis high risk state AND cortisol; biomarkers AND psychosis high risk state AND oxidative stress; biomarkers AND psychosis high risk state AND redox dysregulation; biomarkers AND antipsychotics AND inflammation; biomarkers AND antipsychotics AND stress-response; biomarkers AND antipsychotics AND cortisol; biomarkers AND antipsychotics AND oxidative stress; biomarkers AND antipsychotics AND redox dysregulation.

**Figure 1:**
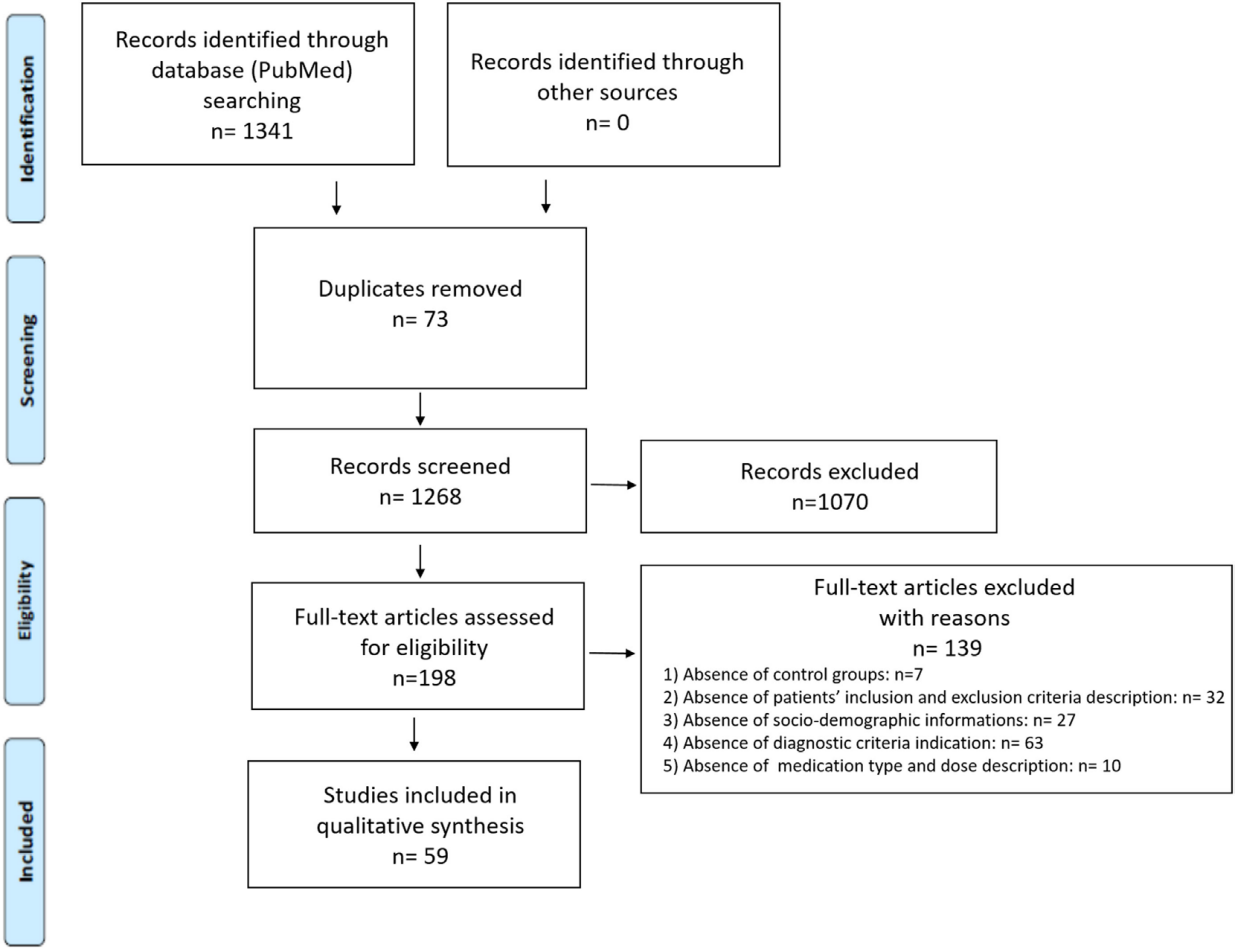
PRISMA flow diagram.

A total number of 1,341 records was obtained with 73 record duplications, which were removed from further screening steps. The following inclusion criteria were applied for the next screening (based on titles and abstracts) of the remaining records (1,268): (1) language of publication (only publication written in English were considered); (2) type of publication (only original research articles, reviews and meta-analysis were considered); (3) subjects of the study (only studies on humans were considered); (4) source of samples (only data obtained from blood samples and derivatives were considered). Based on these criteria, 1,070 records were excluded and 198 full-text articles were assessed for eligibility. We further screened them excluding works (total number 139) which: (1) did not include control group subjects; (2) were missing of a specific description of patients’ inclusion and exclusion criteria: (3) did not report sociodemographic informations for the included patients and controls; (4) did not clearly indicate the diagnostic criteria used for the identification of the considered psychotic stage; (5) did not include a clear indication of the medication type and related doses. Thus, the final number of studies included in the qualitative synthesis was 59 (a PRISMA diagram is provided in Figure 1). The total number of references reported in the bibliography of this review also includes additional publications cited in the Introduction section and in the introducing paragraphs of the other sections of this manuscript.

## Inflammation and Stress Response Biomarkers in Blood and Its Derivatives

Recent data have indicated a pathogenetic link between blood alterations and the psychotic disorder spectrum (3). Blood is considered the most easily accessible biological sample during any stage of the disease (4). This has justified significant efforts in trying to determine blood molecular correlates of schizophrenia and psychosis for early therapeutic interventions (5). In this context, data derived from drug-naive patients, subjects at ultra high risk of psychosis or at first psychotic episode, have been considered extremely precious. Several findings in this field have been published, especially regarding blood biomarkers of inflammation and stress response.

### Inflammation Biomarkers

Over the past few years, there has been an increasing interest in researches focused on the pathogenetic role of inflammation in the development of psychosis and schizophrenia. Interesting data came from some epidemiological studies reporting that increased levels of maternal C-reactive protein, IL-8, and TNFα during childhood are associated to an elevated risk of developing a psychotic state (6). With respect to this point, early life exposure to a chronic inflammatory state, together with a genetically determined impairment of the immune system, have been shown to strongly affect brain development, inducing a vulnerability, which could finally result in evident early psychotic symptoms (7). However, most of the papers focused on this subject lack from clear explanations about the primary cause of this state of increased inflammation.

#### Cytokines

IL-6 and related polymorphisms are the blood inflammatory biomarkers mostly shown as crucial players in the pathogenesis of schizophrenia. Information on their possible role as biomarkers in different psychosis stages and in the prediction of the response to antipsychotics are detailed in Table 1. Data related to other cytokines (IL-1, IL-2, IL-4, IL-10, TNF-α, INF-γ, IL-17, and IL-8) are also reported in the same table.

**Table 1:** Summary of inflammation-related biomarkers.

Inflammation biomarker	Findings	Type of paper and reference
IL-6 and its polymorphisms	– Increased in first psychotic patients – Increased in relapsed patients – Normalization after antipsychotic treatment	Meta-analysis (8)

	Marker of transition from the “risk state” to clinically evident schizophrenia	Original research (9)

	– Positive correlation between increased IL6 levels and an insidious onset of the disease – Positive correlation between increased IL6 levels and a longer duration of total illness – Positive correlation between increased IL6 levels and a more significant deterioration of mental state during the chronic phase – Associated with a major severity of positive symptoms	Original research (10)

	Increased in schizophrenic patients	Original research (11)

	Increased levels in antipsychotic non- responder patients at onset and after 12 weeks of treatment	Original research (12)

	Decreased levels in patients who positively respond to antipsychotic medication	Meta-analysis (13)
		Original research (14)
		Original research (15)
		Original research (16)

	No alterations of serum levels in first psychotic episode patients	Original research (17)

	Increased mRNA levels in leukocytes of first psychotic episode patients	Original research (17)

IL-1	Decreased levels of IL-1ß in first psychotic episode patients and normalization after antipsychotic treatment	Original research (18)
	Increased levels of IL-1ß and IL-1α in first psychotic episode patients	Original research (17, 19)
	Increased levels of IL-1ß in adolescents with acute psychotic episodes	Original research (20)
	Decreased levels of IL-1α after antipsychotic treatment	Original research (18)
	Increased IL-1α mRNA levels in leukocytes of first psychotic episode patients	Original research (17)

IL-2	No changes in patients at ultra-high risk state	Original research (21)
	No changes in first psychotic episode patients	Original research (22)
	Increased in drug-naïve first psychotic episode patients	Original research (23)
	Decreased levels after antipsychotic treatment	Original research (18)

IL-4	No changes in patients at ultra-high risk state	Original research (21)
	– No changes in first psychotic episode	Original research (22)
	– Correlation with an early onset of psychosis	Original research (22)
	– Increased levels in first psychotic episode patients and normalization after antipsychotic treatment	Original research (18)

IL-10	No changes in patients at ultra-high risk state	Original research (21)
	No changes in first psychotic episode patients	Original research (22)
	Increased levels in first psychotic episode patients	Original research (24)
	Correlation with an early onset of psychosis	Original research (22)

TNF-α	No changes in patients at ultra-high risk state	Original research (21)
	No changes in first psychotic episode patients	Original research (22)
	Increased levels in first psychotic episode patients (serum and mRNA levels in leukocytes)	Original research (17, 24)

INF-γ	No changes in patients at ultra-high risk state	Original research (21)
	No changes in first psychotic episode	Original research (22)
	Decreased levels after antipsychotic treatment	Original research (18)
	Increased levels in antipsychotic non- responder patients at onset and after 12 weeks of treatment	Original research (12)

IL-17	Decreased in patients at ultra-high risk state	Original research (21)
	No changes in first psychotic episode	Original research (22)

IL-8	Decreased levels after antipsychotic treatment	Original research (18)
	Increased levels in adolescents with acute psychotic episodes	Original research (20)
	Increased serum levels in first psychotic episode patients	Original research (17)

Few previous publications have examined the validity of other cytokines in identifying subjects at high risk who finally converted to psychosis from those who did not. In an interesting recent study of Föcking and Colleagues, the levels of 40 neuroinflammation biomarkers were measured in “at-risk mental state” subjects who after transitioned to psychotic disorder and compared to the ones of subjects who did not. In this work, authors provided preliminary evidence of an association between elevations in the baseline plasma levels of the inflammatory marker IL12/IL23p40 and the transition from “at-risk mental state” to a clinically evident psychotic disorder (25).

#### Other Inflammation Biomarkers

Very interesting data have been derived from patients with first psychotic episode. Indeed, several recent studies raised the hypothesis that an enhanced inflammatory state may play a crucial role in the earliest stages of psychosis (26). In particular, a 3-month longitudinal study has examined the pathogenetic link between increased inflammation and metabolic changes in 53 patients with a diagnosis of first psychotic episode, showing a significant correlation of increased high-sensitivity C-reactive protein and enhanced triglyceride levels but not gluco-metabolic parameters (27). Interestingly, a population-based longitudinal study, aimed at investigating a possible relation between childhood atopic disorders (associated to increased serum inflammatory markers) and the risk of psychotic experiences, showed that atopic disorders in children may determine an increased risk of developing a psychotic experience during adolescence (28).

#### Inflammation As Possible Protective Component?

Although literature shows quite solid data on the pathogenetic link between increased inflammatory state and psychosis, some studies reported opposite outcomes, attributing, instead, to inflammation a protective role against the risk to develop a psychotic state. In an interesting work, carried on plasma and peripheral blood mononuclear cells of 85 subjects with a clinical diagnosis of first psychotic episode, Authors claimed that some specific markers of inflammation, such as 15d-prostaglandin-J2, may play the role of “protective” biomarkers (29). These contrasting results about the role of neuroinflammation in psychosis and schizophrenia might be explained by the presence of several confounding factors. Among them, the most important one is related to the effects of antipsychotic medication, which is significantly associated to increased risk of weight gain and development of metabolic disorders, characterized “*per se*” by increased inflammation (30). However, observations derived from some meta-analyses and original works, performed by using peripheral blood samples of drug-naive schizophrenic patients, have clarified the existence of a treatment-independent association between increased inflammation and this mental disorder (31). Other important potentially confounding factors include smoking, alcohol, and use of illicit psychoactive compounds. Nonetheless, studies that classify psychotic patients and respective controls according to these variables, with a consequent analysis of the statistical impact of these confounding elements, are still very poor (32) and contrasting results have been obtained from them.

### Stress Response Biomarkers

Several lines of evidence have pointed towards a pathogenetic link between stressful events in life, individual vulnerability, and the development of psychotic conditions, in particular first psychotic episode (33–35). Although the methodological validity of most of the studies focused on this subject, it should be adequately taken into account that stress response biomarkers (in particular, cortisol) have been proposed for many other psychiatric conditions [such as autism (36), anxiety (37), bipolar and post traumatic stress disorders (38, 39)] and non-psychiatric illnesses [such as cardiovascular (40), proliferative (41) and gastrointestinal disorders (42)].

#### Stress Response Biomarkers in Ultra-High Risk and First Psychotic Episode Patients

It has been reported that “Ultra-High Risk” patients, as well as subjects with a clinical diagnosis of first psychotic episode, showed increased levels of emotional reactivity with an enhanced subjective perception of stress in response to adverse life events (43–45). This has mainly been attributed to alterations of the HPA axis functioning (46, 47), in particular, to its hyperactivity (48, 49) and to consequent elevation in cortisol release (50–52). Cortisol levels in psychotic patients and in subjects at high risk to develop this mental disorder have been mainly quantified in other biological samples rather than blood, such as saliva (53–55) or urine (56, 57). Thus, a minor number of studies, aimed at identifying cortisol levels as a valid biomarker for psychosis, has been performed using blood samples. Among them, an interesting study quantified serum cortisol levels in 60 schizophrenic patients compared to 70 healthy first-degree relatives and 60 healthy volunteers, showing higher cortisol levels in the serum of schizophrenic patients compared to the other experimental groups. Interestingly, healthy first-degree relatives showed significantly increased serum cortisol when compared to the healthy subject group (58). A recent study by Sun and collaborators performed on blood samples collected from 15 healthy subjects controls and 13 schizophrenic patients showed increased basal level of cortisol in this group, also persisting after 8 weeks of clozapine treatment (59).

#### Stress Response Biomarkers for the Prediction of the Pharmacological Response

Blood cortisol levels have been proposed as valid biomarker and predictor of the pharmacologic response in patients with psychotic disorders. Indeed, Babinkostova et al. evaluated blood cortisol levels of 60 schizophrenic patients compared to 40 age and sex matched healthy subjects, both at baseline and after 3 and 6 weeks of treatment with neuroleptics. Serum cortisol levels were found elevated both in schizophrenic patients and in antipsychotic treatment responders compared to non-responder patients (60). In another work, evaluating cortisol levels in 31 healthy controls, 48 treatment-responder, and 59 treatment non-responder schizophrenic patients, increased cortisol concentrations were detected in the group of responders with respect to healthy subjects. Only a trend in cortisol levels elevation was observed in non-responder schizophrenic patients compared to controls, while no differences were detected in treatment responders and non responders (61). Interestingly, when plasma cortisol levels were analyzed at the inpatient status, in order to eliminate the effect that hospitalization/acute relapse may have on the stress response, a significant difference in plasma cortisol was found in treatment-responder patients with respect to non responders, although a significant difference between inpatient and outpatient treatment-non responder schizophrenic subjects was not observed (61).

However, other reports did not replicate these findings. Indeed, a study of Simsek and collaborators investigated serum cortisol levels in adolescent patients with a clinical diagnosis of first-episode early onset schizophrenia, detecting no significant differences in serum cortisol between adolescent psychotic patients and controls (62). In the same line, Tobolska and co-workers failed to identify differences in cortisol levels measured on blood samples collected from 10 schizophrenic patients compared to 38 healthy individuals, while finding significant differences of cortisol levels in other biological samples (i.e., saliva and urine) derived from the same patients (55). These observations were also supported by another recent report describing a decreased resilience capacity in schizophrenic patients which, however, did not correlate with alterations in blood stress-related biomarkers, such as cortisol and ACTH (63).

## Redox Imbalance Biomarkers

Several publications have identified redox imbalance as a crucial player in the pathogenesis of psychosis. Most of the data on this subject have been obtained on both pharmacologic and non-pharmacologic rodent models of this mental disorder (64–71). Increasing research interest is going toward the possibility to translate the findings obtained on animal models toward humans. Indeed, a consistent number of works are focusing on the measurement of reactive oxygen species amount, oxidative damage markers, and antioxidant defense functioning in different types of samples derived from psychotic medicated and unmedicated patients (72).

Table 2 reports an overview of the most relevant findings about the use of redox status-related biomarkers both in different stages of psychosis development and as predictors of response to medication.

**Table 2:** Summary of redox status-related biomarkers.

Condition	Biomarkers	Source of samples	Association (yes/no)	Reference
Psychotic risk state	Thiobarbituric acid reactive substance	Serum	Yes	(73)
	MDA-modified LDL	Blood	Yes	(26)
	Polymorphism of the d-aminoacid deoxide dismutase activator gene	Blood	Yes	(74)

First psychotic episode	Free radical amount	Blood	No	(22, 75)
	DNA damage	Blood	No	(22, 75)
	SOD	Blood	No	(76)
	Glutathione peroxidase	Blood, plasma, erythrocytes	No/Yes	(76, 77)
	8 OhdG	Blood	No	(76)
	Lipid hydroperoxides	Blood, plasma, erythrocytes	No/Yes	(76, 77)
	Nitric oxide-derived metabolites	Blood	No	(76)
	Oxidation protein products	Blood	No	(76)
	Total radical-trapping antioxidant parameter	Blood	No	(76)
	Paraoxonase I	Blood	No	(76)
	Thioredoxin-1	Plasma	No	(76)
	Altered redox status	Plasma, erythrocytes	No	(78, 79)
	Total antioxidant status	Blood	Yes	(80)
	Total glutathione levels	Blood	Yes	(80, 81)

Response to antipsychotic therapy	–	–	–	

### Redox Dysregulation Biomarkers for the Psychosis Risk State and for First Psychotic Episodes

Interesting findings have been obtained in human subjects about the possibility to consider redox dysregulation as a valid biomarker of the risk state to develop a psychotic condition. In this context, Pedrini and Collaborators have evaluated oxidative stress markers and cytokine levels in serum samples collected from subjects at high risk of psychosis, with respect to sex and age matched healthy subjects, showing increased thiobarbituric acid reactive substance and IL-6 in patients at risk for psychosis, compared to controls (73). The North American Prodrome Longitudinal Study, an 8-site observational work, including 765 clinical high-risk and 280 demographically similar healthy subjects, aged between 12 and 35, has focused on the identification of the psychosis conversion predictors. Importantly, in this study, blood levels of malondialdehyde-modified low-density lipoprotein, an oxidative stress biomarker, were found increased in high-risk subjects with respect to controls and positively correlated to the conversion toward a clinically evident psychotic state (26). In support to these observations, a genetic polymorphism of the d-aminoacid deoxide dismutase activator gene, modulating antioxidant pathways, have been associated with the clinical transition from the high-risk state to first psychotic episode in adolescent subjects (74). A considerable number of studies have been published on the possible identification of increased oxidative stress or decreased antioxidant defense as reliable biomarkers of the first psychotic episode. However, contrasting results are reported in these works. Indeed, a very recent study by Şimşek and collaborators, comparing free radical levels and the derived DNA damage in untreated first psychotic episode adolescents with sex and age matched control subjects, did not show any differences in specific markers of redox regulation (including superoxide dismutase, glutathione peroxidase, and 8-hydroxy-2-deoxyguanosine) between experimental groups (75). In the same line, another recent study showed no significant association among oxidative stress markers (such as lipid hydroperoxides, nitric oxide-derived metabolites, and advanced oxidation protein products), antioxidant biomarkers (such as total radical-trapping antioxidant parameter and paraoxonase 1), and clinical data in a population of 51 drug naive first psychotic episode patients (76). An interesting work performed by the group of Owe-Larsson aimed at investigating whether plasmatic levels of thioredoxin-1 could be used as a valid biomarker to distinguish patients at their first psychotic episode from chronic schizophrenics and non-psychiatric subjects. In this study, authors claimed that thioredoxin-1 levels cannot be considered an accurate and reliable biomarker to identify first psychotic episode patients and to discriminate patients suffering from psychosis or schizophrenia from individuals not affected by a mental disorder, although a more pronounced altered redox state was observed in psychotic subjects (82). In the same line, Parellada and Collaborators did not find any association between oxidative stress markers and the prediction of 2-year functional and clinical outcomes in a cohort of children and adolescents with a first episode of psychosis (78). Similar conclusions on the redox status as an unreliable biomarker related to first psychotic episode have been also reached by the group of Sarandol. Indeed, in an elegant work performed on 29 first psychotic episode patients and 25 control subjects, although the finding of an increased oxidative stress in psychotic subjects, 6 weeks of antipsychotic treatment failed in reestablishing the physiological redox state (79). In contrast, basal total antioxidant status and glutathione levels have been described as decreased in patients with early onset first psychotic episode; being the antioxidant status also associated with memory impairment and attention deficits in these subjects (80). Accordingly, a multicenter study, investigating the possible pathogenetic link between redox dysregulation and gray matter alteration in patients at first psychotic episode by magnetic resonance imaging, reported a significant association between lower basal glutathione concentrations levels and decreased volume of left frontal, parietal, and temporal regions as well as gray matter mass alterations (81). Another interesting case-control study, analyzing the plasmatic total antioxidant status and lipid peroxidation in 102 children and adolescents with a clinical diagnosis of first psychotic episode compared to 98 healthy age and sex matched controls, reported decreased antioxidant defense (in term of diminished activity of the glutathione peroxidase system) and elevation in products derived by the lipid peroxidation process in first psychotic episode patients with respect to controls (77).

### Redox Dysregulation Biomarkers for the Prediction of Pharmacological Response

To the best of our knowledge, no specific and detailed data about the possibility to use increased free radical production, oxidative stress-derived damage, and decreased antioxidant status as reliable biomarker of the response to neuroleptics are actually available. Indeed, most of the available reports only focused on the effects of the antipsychotic treatment on the redox status of patients suffering from psychosis and schizophrenia at different clinical stages of the disease (51), as well as the possible use of antioxidant compounds, such as the N-acetylcysteine, in the treatment of the psychotic condition, especially at its very early stages (83–85). Thus, the identification of redox-related biomarkers to be used to predict and monitor the individual response to antipsychotics might represent an interesting direction for future research.

## Conclusion

Identifying a biomarker for psychosis would certainly lead to several advantages, such as the possibility of an early diagnosis, of a stratification of patient population as well as of a progression toward personalized therapies. Indeed, with respect to this last point, several extrinsic and intrinsic factors (such as genetic aspects, environment and neuronal alterations at both cellular and structural levels) may account for the interindividual variability in the response to the pharmacological compounds commonly use both to treat a frank psychotic state or to slower the progression toward this mental disorder. Another crucial benefit will also consist in the possible combination of individual genetic data with specific biomarkers in order to more effectively treat single cases of psychosis. Furthermore, even if a proposed biomarker could not be considered reliable for a group of patients, it might be predictive, or even associate, with specific aspects of the disease such as its severity or particular symptoms (e.g., positive versus negative symptoms of psychosis) in a single subject, probably because his/her unique genetic asset.

However, more than in other medical fields, the efforts of the scientific community have not been yet translated in what expected. Indeed, so far, most of what is considered a biomarker candidate for psychosis is instead only a potential biomarker. That said, important questions arise: if, for other medical fields, research has succeeded in identifying clinical applicable biomarkers, why it should not happen for psychiatric disorders and, in particular, for psychosis? Thus, which major differences exist with respect to other psychiatric conditions and to non-psychiatric pathologies? A possible attempt to answer to these questions should consider the complex nature of mental disorders with respect to other non-psychiatric conditions, requiring a more global approach in which the different pathogenetic elements should be adequately taken into account. Furthermore, with respect to other psychiatric disorders, the possibility to follow-up psychotic patients in longitudinal studies, which will provide a tool to overcome some time-related limitations in the field of biomarker research, is challenging given the high rates of noncompliance (especially to medication) of psychotic patients. Another important aspect to consider in psychosis is the presence of several comorbidities that significantly decrease the possibility that an identified biomarker candidate can be routinely used for the clinical practice. Thus, so far, available literature clearly indicates the absence of biomarkers for psychosis, in particular, for the identification of the ultra high-risk state, first psychotic episodes, and pharmacological response. Furthermore, the absence of a deep and comprehensive understanding of all the different players involved in psychosis pathogenesis has been highlighted leading to several speculations about the molecular mechanisms underlying the onset and progression of psychotic symptoms and the increasing “bad habit” to directly translate data obtained from biomarker research in animal models of this mental disorder to humans. Therefore, future research in this field will certainly need a more global overlook toward psychosis pathogenesis but, mostly, a more cautious approach in considering and diffusing obtained results.

## Author Contributions

Writing manuscript, SS. Revising manuscript content, LT. Approving final version of the manuscript, SS and LT.

## Conflict of Interest Statement

The authors declare that the research was conducted in the absence of any commercial or financial relationships that could be construed as a potential conflict of interest.

## Abbreviations

IL-8, interleukin 8; TNF-α, tumor necrosis factor alpha; IL-6, interleukin 6; IL-12, interleukin 12; IL-23p40, interleukin 23 p40; INF-γ, interferon gamma; IL-10, interleukin 10; Th17, T helper 17; HPA stays for hypothalamic-pituitary-adrenal axis; ACTH, adrenocorticotropic hormone; TBARS, thiobarbituric acid reactive substance; NAD, nicotinamide adenine dinucleotide.
